# Diet, Activity, and Overweight Among Preschool-Age Children Enrolled in the Special Supplemental Nutrition Program for Women, Infants, and Children (WIC)

**Published:** 2006-03-15

**Authors:** Jennifer A Nelson, Kathleen Carpenter, Mary Ann Chiasson

**Affiliations:** County of San Diego Health and Human Services Agency, Community Epidemiology; At the time of the study, Ms. Nelson was affiliated with the Medical and Health Research Association of New York City, Inc, New York, NY; Medical and Health Research Association of New York City, Inc (MHRA) Neighborhood Women, Infants, and Children (WIC) Program, New York, NY; New York, NY

## Abstract

**Introduction:**

Overweight is affecting children at younger ages and in increasing numbers, putting them at risk for a lifetime of chronic disease. Consumption of unhealthy foods and time spent watching television have increased concurrently.

**Methods:**

Parents of 526 children aged 2 to 4 years old enrolled in the Special Supplemental Nutrition Program for Women, Infants, and Children (WIC) answered questions about their children's food and beverage consumption, television-viewing and computer time, and physical activity. The children's height and weight measurements were collected from administrative records. Crude and adjusted odds ratios were calculated to test for associations between demographic, consumption, and activity variables and overweight or at risk of overweight (body mass index ≥85th percentile for age and sex).

**Results:**

Of the participants, 38% of the children were overweight or at risk of overweight. Hispanic and white children were twice as likely as black children to be overweight or at risk of overweight. Fifty-eight percent of the children drank more than one and 30% drank more than two 8-oz servings of fruit juice per day. The children who drank more than one serving of nonjuice fruit drink per day (30%) had increased odds of being overweight or at risk of overweight. On average, the children spent more than twice as much time watching television and using computers as they did engaging in physical activity. In multivariate analyses, race and ethnicity as well as physical activity were associated with being overweight or at risk of overweight.

**Conclusion:**

Efforts to improve nutrition and prevent overweight in children should focus on the parents of infants and toddlers and provide them with anticipatory guidance on physical activity for young children and nutrition and food transitions.

## Introduction

Overweight is quickly becoming one of the defining public health problems of childhood. The severity of the situation is underscored by the younger ages at which overweight is affecting children, putting them at risk for a lifetime of health problems such as type 2 diabetes and cardiovascular disease. Nationally, according to the 1999–2002 National Health and Nutrition Examination Survey (NHANES), the prevalence of overweight among children aged 2 through 5 years is 10% (1), which is double the rate reported two decades ago (2). When children who are at risk of overweight are included, the prevalence is 23% (1). In New York City, NY, a 2003 survey of public school children found that 48% of kindergarten children were already overweight or at risk of overweight (3).

The weight increases have largely coincided with changes in diet and consumption patterns. Studies using national data sets have reported increases in portion sizes and energy intake among adults and children for many commonly consumed food items, particularly pasta; salty snacks; and juice, soda, and other sugar-sweetened beverages as well as an increase in the percentage of children consuming these items (4-6). Increased consumption is only half of the energy imbalance responsible for weight gain and overweight. Researchers have begun documenting the amount of time children spend watching television or videos (7-10); television viewing has been associated with overweight in children in multiple studies (7,9,11-14). Furthermore, although no good data exist on physical activity trends in children over time and a consensus has not been reached on how to define and measure physical activity, some evidence suggests that many children are insufficiently active (7,11,15). Data on physical activity in very young children are particularly scarce.

Childhood overweight is clearly a complex issue with many related causes, including biological factors, environment, diet and nutrition, and activity level. Many studies have assessed various factors separately, but few have examined their relationship to one another and overweight. The high prevalence of overweight among preschool-age children suggests that greater attention should be paid to this population so that we can increase our understanding of the problem and ways it might be addressed. The Special Supplemental Nutrition Program for Women, Infants, and Children (WIC) serves many children at this crucial age. In previous work, the authors found a high prevalence of overweight in a New York City population of children enrolled in WIC, with particularly high levels among Hispanic children (16). In our study, we sought to identify some of the food and beverage consumption patterns and television-viewing and physical activity habits in this population, as well as ways the patterns relate to each other and to overweight.

## Methods

### Setting and survey administration

The Neighborhood WIC Program, administered by the Medical and Health Research Association of New York City, Inc (MHRA), is the largest WIC program in New York State, serving more than 45,000 women, infants, and children each year at 18 sites throughout New York City. To examine nutrition, physical activity, and television-viewing and computer use patterns (screen time) among children in this population, we conducted a survey of all parents and guardians who brought a child aged 2 to 4 years to one of 16 WIC centers for a certification visit over the course of 5 to 10 days during October 2003. WIC requires that a primary caretaker with knowledge of the child's habits bring the child to the center for certification visits; 90% of the people who completed the survey were the mothers of the children, so the primary caretakers are referred to as *parents* throughout this article. WIC staff members asked parents to fill out a 1-page anonymous questionnaire while waiting to receive services. Nutritionists provided assistance as needed with filling out the questionnaire, ensured that the questionnaire was complete, and recorded each child's height, weight, age, and sex on the form. The questionnaire was available in English, Spanish, Chinese, and Russian; forms were translated by WIC staff with the appropriate language skills.

Based on WIC administrative data for the period, we estimate that we had an 80% response rate (n = 526). Because of our reluctance to disrupt program activities and our reliance on staff members to distribute and collect the surveys, we were unable to track reasons for lack of response; however, staff members indicated that very few parents refused. In some instances, the high volume of clients prevented distribution or completion of questionnaires. This type of lack of response was likely to have been randomly distributed among the population surveyed. A demographic comparison of the sample to administrative data proved the sample to be representative of the overall population (Table 1).

### Variables

The survey included questions about parental perception of the child's size, whether the child had been diagnosed with asthma, the child's race and ethnicity, and the family's country of origin. Parents were asked to report which type of milk their children drank most often, how many servings of fruits and of vegetables their children ate on average each day or each week; how many times per day or week their children ate snack foods such as chips, pretzels, cookies, or candy; and how many minutes or hours per day their children spent playing actively or exercising and watching television or using a computer. Parents were also asked the number of cups per day or week that their children drank of plain milk, flavored milk, nonjuice fruit drinks, fruit juice, water, and soda or sweetened iced tea. The questions about physical activity and screen time were adapted from an NHANES instrument (17). The format and content of the diet-related and other questions were similar to those routinely asked of WIC clients as part of the intake process. A small pilot test was conducted in one WIC site to ascertain that our questionnaire was relevant and appropriate for the population to be surveyed.

Calculations were made to create continuous consumption and activity variables with the units of *servings per day* or *minutes per day*. Dichotomous variables were also constructed for each measure, using recommended consumption and viewing or activity levels as cutoff points whenever possible. Body mass index (BMI) was calculated from height and weight and compared with the age- and sex-specific reference standards developed by the National Center for Health Statistics at the Centers for Disease Control and Prevention (CDC) (18). A child with a BMI at the 85th percentile or higher was considered *overweight* or *at risk of overweight*.

### Statistical analysis

A total of 593 questionnaires were returned; 67 were excluded because of missing or out-of-range data on key variables (e.g., age), leaving an analytic sample of 526. Actual sample sizes for individual analyses vary because of missing data (including many inconsistent and implausible responses) for individual variables. Missing and implausible responses were excluded from analyses at the variable level to maximize the sample size for each bivariate comparison. Exploratory analyses revealed that excluding data at the variable level rather than the respondent level did not appreciably change the results; in addition, respondents with one or more implausible responses were not significantly different from those with none.

Medians were used to determine average daily consumption of foods and beverages, average screen time, and average active playtime. Exploratory tests for an association between BMI percentile and consumption and between BMI percentile and activity using continuous variables were also conducted. However, because of the clustering of responses at a few values and the belief that categorical comparisons would provide results with greater relevance for public health practice, we have presented only the categorical data analysis findings. Chi square tests using the continuity correction for two-by-two tables were used to assess associations among demographic, consumption, and activity variables. Bivariate odds ratios with 95% confidence intervals were calculated to establish the relationships between demographic, consumption, and activity variables and overweight and risk of overweight. Multivariate analyses using logistic regression were conducted to compute adjusted odds ratios and assess whether associations that were significant at the bivariate level remained significant when controlling for demographic and other variables. Demographic and health data from the survey were compared with data on children making initial visits to MHRA WIC sites in 2003 to assess whether the sample was representative of the larger MHRA WIC population.

Data analysis was performed using SPSS version 11.5.1 (SPSS Inc, Chicago, Ill). The research protocol was approved by the MHRA Institutional Review Board.

## Results

### Sample characteristics

As shown in Table 1, the sample was composed of more boys than girls and more 2- and 3-year-olds than 4-year-olds. Most children were either Hispanic or black. The proportions were similar to those of the larger MHRA WIC population. Most were from families whose origin was outside of the United States and Puerto Rico. Thirteen percent of the children had been diagnosed with asthma, and many were overweight (21%) or at risk of overweight (17%). Ninety-seven percent of parents reported that their children were just the right size or underweight (Table 1), including 93% of parents of overweight children and 95% of parents of children at risk of overweight (data not shown).

### Beverage and food consumption

Water and plain milk were the beverages most frequently consumed by the children (Table 2). Seventy-nine percent of the children drank whole or 2% milk (data not shown). Fruit juice consumption was high (with a median of two servings per day), and a substantial proportion of the children also consumed flavored milk (e.g., chocolate) and nonjuice fruit drinks. Although soda consumption was considerably lower, more than half of the children consumed some soda or sweetened iced tea during the average week.

Fruit and vegetable consumption was far less than the recommended amounts (Table 2). On average, the children consumed one serving of each every day (Table 2), and only 12% ate at least five servings per day (data not shown). Snack food consumption, although considerably lower than fruit and vegetable consumption, is a concern for a portion of this population; 40% ate a snack of chips, pretzels, cookies, or candy at least once per day (data not shown).

Some demographic differences in consumption were found, but few were statistically significant (data not shown). Consumption of soda and snack foods increased with age; by age 2, 44% of the children were consuming at least one unhealthy snack food or soda every day, a proportion that increased to 57% by age 4 (*P* = .10). Hispanic children were far less likely than other children to eat snack foods every day. Thirty-one percent of Hispanic children ate a snack food at least once per day, compared with almost 50% or more of non-Hispanic children (*P* < .001), and 9% of Hispanic children ate a snack food more than once per day, compared with almost 20% or more of non-Hispanic children (*P* < .001). Country of family origin was significantly associated with food but not beverage consumption. Children whose families were from the United States or Puerto Rico were more likely to eat a snack food at least once per day (*P* = .01), more likely to eat more than two servings of vegetables per day (*P* = .04), and less likely to eat more than one serving of fruit per day (*P* = .02).

### Physical activity and television

On average, children spent more than twice as much time watching television or using a computer (screen time) as they did playing actively or exercising (Table 3). Although no differences in screen time or time spent playing actively were found based on age or sex, some differences based on other characteristics were found (data not shown). In a comparison of black and Hispanic children only, which were the two largest groups in the sample, we found that a greater proportion of black (50%) than Hispanic (34%) children spent at least 1 hour daily playing actively (*P* = .01). In addition, 34% of children whose families were from the United States or Puerto Rico had more than 2 hours per day of screen time, compared with 24% of children whose families were from outside of the United States (*P* = .04). Children of families from the United States also spent more time being physically active; 53% spent at least 1 hour per day playing actively, compared with 32% of other children (*P* < .001). Asthma was also associated with screen time; 43% of children with asthma spent more than 2 hours per day watching a television or computer screen, compared with 24% of children without asthma (*P* = .001). However, asthma was not associated with time spent in active play.

Children who spent at least 1 hour per day playing actively were also more likely to consume more than one nonjuice fruit drink per day (*P* = .01) and to have more than 2 hours daily of screen time (*P* < .001). In turn, 39% of children who had more than 2 hours per day of screen time drank more than one nonjuice fruit drink per day, compared with 28% of children who had less screen time (*P* = .03).

### Overweight and at risk of overweight

Almost 40% of the children were overweight or at risk of overweight. Bivariate analyses (Table 4) revealed significant differences by race and ethnicity. Hispanic and white children were two times as likely as black children to have a BMI at or higher than the 85th percentile. The only diet variable associated with increased odds of being overweight or at risk of overweight was consuming more than one nonjuice fruit drink per day (*P* = .046). Screen time of more than 2 hours per day and active playtime of less than 30 minutes per day each increased the odds of overweight and at risk of overweight, although the associations were not statistically significant. Compared together as a ratio (screen time/active play time), they were significantly associated with overweight and at risk of overweight.

Multivariate analyses revealed similar associations (Table 4). A logistic regression model was constructed that included age, sex, and the variables with a *P* value of less than .10 in a bivariate test of association (race and ethnicity, asthma, nonjuice fruit drink, and screen time). A physical activity variable was included as well, because insufficient activity has been associated with overweight in other populations. The adjusted odds of Hispanic and white children being overweight or at risk of overweight compared with black children were significantly higher. The bivariate association with nonjuice fruit drink consumption became marginally significant, though the odds ratio remained the same. The association with screen time remained marginally significant, whereas the association with active playtime became stronger and gained significance.

## Discussion

Almost 40% of the sample — urban, preschool-age children enrolled in WIC — was overweight or at risk of overweight, substantially more than in a national sample (1). Some studies have found low-income children to be at greater risk of overweight (19,20); this study population was composed entirely of low-income children, which may explain the divergence from NHANES data. Data from the Pediatric Nutrition Surveillance System (PedNSS) show that nationwide, children enrolled in WIC and other maternal and child health programs have only a slightly lower prevalence of overweight (14.7%) and risk of overweight (15.7%) than found in this study (21). In addition, our sample may include a larger percentage of overweight children because of the programmatic requirements of WIC, in which overweight is one of the nutritional risk factors that make a child eligible for the program. However, the proportions are similar to those found among slightly older kindergarten children in a larger New York City study of overweight among schoolchildren (3) and in a study of Hispanic children aged 5 and 6 years old in Chicago (22).

The prevalence of overweight and risk of overweight was particularly high among the Hispanic children in this population, again compared with the prevalence reported in the NHANES data but not in the PedNSS data. In contrast, non-Hispanic black children had the lowest prevalence of overweight and at risk of overweight in the study population and were the most similar to national data. Although our findings need to be corroborated by other studies in different locations, they suggest that the public health community should be cautious in making assumptions about the risks and needs of minority children. Some studies have found higher rates of overweight and more pronounced increases in overweight among Hispanic and African American children (1,23), but some evidence suggests that the increases vary by age, income, and other factors (24). For example, our findings show that nutrition and activity risk factors vary as well. Hispanic children in our study ate substantially fewer snack foods than other children, yet more of the Hispanic children were overweight. Black and Hispanic children drank considerable amounts of nonjuice fruit beverages, a potential risk factor for overweight, yet had different levels of overweight. Our findings suggest that the interaction of race and ethnicity, age, and poverty (and likely other factors) with nutrition and physical activity habits account for differences in prevalence of overweight.

Other than race and ethnicity, the strongest predictors of overweight in our study were a low level of physical activity and a large amount of screen time, particularly relative to active playtime. Data on the relationship between overweight and physical activity among very young children are scarce, but one study found overweight preschool boys to be significantly less active than boys who were not overweight (25). In addition, the combined adverse effects of television watching and low levels of physical activity on body fat were found to increase over time in a longitudinal study by Proctor et al (12). The relationship among physical activity, screen time, and overweight is not straightforward. It seems reasonable to expect that spending many hours in front of a television or computer screen and not enough time engaged in physical activity could be independently associated with overweight, but screen time does not necessarily predict physical activity. In fact, we found that higher levels of physical activity were associated with more screen time. It may be that having an appropriate balance between screen time and physical activity is most important, which may help explain why we found a stronger association with overweight when we assessed it with the ratio between screen time and active play time and why the association between active play and overweight strengthened when accounting for additional variables in multivariate analyses. The relationships warrant additional investigation.

On average, we found that the children in our sample spent much more time in inactive pursuits than active ones. Many of the children in our study exceeded the 2-hour television limit recommended by the American Academy of Pediatrics (AAP) (26) and failed to meet the 60-minute physical activity recommendation in the *2005* *Dietary Guidelines for Americans* (27), with many not even reaching 30 minutes per day. The poor, urban neighborhoods where many of these children live, with their dearth of safe, open play spaces, may contribute to this imbalance (28-30).

We did not find the sex difference in physical activity levels noted by other researchers (11,15), which may be attributable to the very young age of the children in our sample. Sex differences in learned behaviors may develop as children get older and spend more time interacting with other children. In addition, physical activity levels can be difficult to assess among very young children (31), and our measure — a single question about time spent in activity — may not have adequately addressed types of physical activity in which sex differences among this population could be detected. Studies that have found sex differences in activity levels among preschool-age children have tended to use multiple measures or more fully assess intensity of as well as time spent in physical activity (29,32).

Some researchers have found not only that excessive television viewing is linked to overweight but also that correlations exist between watching television and increased energy intake (7) and soft drink consumption (33), effectively doubling the health risks related to television. In our study, more than 2 hours per day of screen time was associated with drinking more than one 8-oz serving of nonjuice fruit drink per day. The high consumption level of this type of beverage is especially worrisome because nonjuice fruit drinks often seem healthy but actually contain large amounts of sugar and have little or no nutritional value. In fact, multiple studies have shown an association between consumption of sweetened beverages and overweight in various populations of children and adults (34-37).

We found consumption levels of sweetened beverages among very young children to be high, a finding corroborated by other studies. Rampersaud et al found that mean consumption of nonjuice fruit drinks and soda exceeded consumption of 100% fruit juice by 5 years of age and exceeded milk consumption by 13 years of age (38). Juice and sweetened beverages have been found to displace milk in children's diets when they are as young as 15 to 24 months of age (39). Research has shown that beverages are significant contributors to young children's nutrient and energy intake (39); however, parents may consider liquid consumption to be harmless and overlook unhealthy qualities such as a high sugar content.

Ponza et al demonstrated that toddlers participating in WIC were more likely than other toddlers to consume greater amounts of juice, fruit drinks, and sweetened beverages, so the problem in this population may warrant particular attention (40). In addition to highly sugared nonjuice fruit drinks, the WIC-enrolled children in our study consumed large amounts of fruit juice — a median of two 8-oz servings of fruit juice per day, with a sizeable proportion consuming even more, compared with the 4- to 6-oz serving per day recommended by the AAP (41). Parents may consider fruit juice to be a nutritious beverage choice for their children and may not realize that they should monitor the volume consumed.

We found no other significant associations between consumption variables and overweight or risk of overweight, yet the apparent nutritional deficiencies are still a concern. Fruit and vegetable consumption was much lower than the recommended levels; only 12% of the sample consumed 5 servings per day. In addition, 34% of children ate at least one unhealthy snack food per day. The combination of too little fruits and vegetables and too many unhealthy snack foods suggests the need for an intervention. Parents should be encouraged to offer their children fruits and vegetables as nutritious snacks to replace the chips and candy they are now eating.

This study has several limitations. First, our data are cross-sectional, so the directionality of the associations cannot be ascertained. Second, consumption and activity were assessed using parent reports on a self-administered questionnaire, and the format of some of the questions may have been confusing to respondents. In addition, serving sizes can be difficult to assess; written descriptions of beverage but not food serving sizes were provided; amount estimation guides were not used (e.g., cups, ounces). These factors make the findings subject to the usual caveats of self-reported data (in our study, by proxy), such as recall and social desirability biases. However, because considerable variations exist — cultural and otherwise — in the nutritional values assigned to different foods, we cannot make any assumptions about overreporting or underreporting consumption of any particular item. Parents may have equally overestimated or underestimated consumption, so we have no reason to believe that the estimates biased our findings. Third, bias may have been introduced when WIC staff assisted some parents with completing the questionnaire, some of whom (the parents) may have had limited literacy or language skills. We were unable to track such effects at the WIC sites. However, the WIC staff members reviewed all completed questionnaires to identify problems such as missing or inconsistent responses. Fourth, not all aspects of diet, nutrition, and physical activity were measured, making the picture of children's diet and physical activity presented here an incomplete one. Fifth, because the sample was meant to reflect the population of MHRA WIC participants, which is largely Hispanic, only small numbers of some of the other racial and ethnic groups were represented. The relatively low number of white and Asian children in the sample means that findings involving these groups should be considered in light of the sample size. Finally, a substantial amount of data was missing, which reduced the power to detect differences, particularly in the multivariate analyses.

Despite these limitations, our findings have implications for nutrition education and overweight prevention programs. We cannot emphasize enough the high prevalence of overweight and evidence of poor nutrition in our study's group of 2- to 4-year-olds. It is important to set good examples and instill healthy lifetime nutrition and physical activity habits in children at a young age so that we can prevent overweight before it becomes a problem. Parental perceptions warrant attention as well; the parents surveyed in this study did not perceive their children to be overweight — even when their children were overweight — a finding comparable to other studies (42-44). Overweight may be caused by a simple imbalance between consumption and physical activity, but the factors that create that energy imbalance are anything but simple. A complex array of factors interact in various ways among different segments of the population.

A general strategy to address our findings should encourage more physical activity in relation to screen time and promote good nutrition, with particular attention paid to types and amounts of beverages consumed, but the messages may need to be tailored for various populations, and they must begin when children are very young. In our study, many poor nutrition practices were already apparent by age 2 and were worsening by age 4. A study of younger children found a steady increase in the consumption of items such as unhealthy snack foods and soda between the ages of 9 and 11 months and between 19 and 24 months (41). Many dietary changes occur during the first 5 years of children's lives, changes that parents may be ill-equipped to handle. Efforts to improve nutrition and prevent overweight in children should focus on the parents of infants and young toddlers, providing them with guidance on physical activity for young children, nutrition and food transitions, and recognizing overweight and its consequences.

## Figures and Tables

**Table 1:** Characteristics of the Study Population and the Medical and Health Research Association (MHRA) Women, Infants, and Children (WIC) Population, New York City, NY, 2003

**Characteristic**	**Study Population No. (%)**	**MHRA WIC Population^b^No. (%)**
Total^a^	526 (100)	2024 (100)

**Sex**		

Male	264 (55.5)	1024 (50.6)
Female	212 (44.5)	1000 (49.4)

**Age, y**		

2	211 (40.1)	791 (39.1)
3	196 (37.3)	773 (38.2)
4	119 (22.6)	460 (22.7)

**Race or ethnicity**		

Hispanic	282 (55.0)	946 (51.6)
Non-Hispanic black	117 (22.8)	506 (27.6)
Non-Hispanic white	47 (9.2)	174 (9.5)
Asian	40 (7.8)	207 (11.3)
Other and mixed race	27 (5.3)	NA

**Family origin**		

United States or Puerto Rico	134 (29.3)	NA
Other	323 (70.7)	NA

**Asthma**		

Yes	66 (12.8)	NA
No	451 (87.2)	NA

**Weight status**		

Overweight	95 (20.7)	325 (17.1)
At risk of overweight	78 (17.0)	341 (18.0)
Normal	263 (57.3)	1113 (58.7)
Underweight	23 (5.0)	118 (6.2)

**Parental perception of child's weight**		

Just the right size	451 (87.7)	NA
Overweight	16 (3.1)	NA
Underweight	47 (9.1)	NA

a Some subtotals do not equal 526 because of missing data.

b MHRA WIC administrative data, initial visits of 2- to 4-year-olds, 2003. NA indicates WIC data not available.

**Table 2:** Daily Beverage, Snack, and Fruit and Vegetable Consumption Among Children Enrolled in a New York City Women, Infants, and Children (WIC) Program, 2003

**Item Consumed**	**Any Consumed Each Week (%)**	**Median No. of Servings/Day^a^ (Range)**	**Servings/Day^b^ (%)**		

			**≥1**	**≥2**	**≥3**

**Beverage**					

Plain milk	96.7	2.0 (0.1-8.0)	72.2	47.7	22.4
Flavored milk	63.8	1.0 (0.1-8.0)	27.4	9.6	4.2
Fruit juice	95.1	2.0 (0.1-8.0)	58.1	30.3	10.9
Nonjuice fruit drink	68.6	1.0 (0.1-7.0)	30.2	15.7	3.3
Water	98.2	3.0 (0.3-10.0)	73.7	52.6	27.3
Soda or sweetened iced tea	52.5	0.4 (0.1-4.0)	3.7	1.0	0.2

**Food**					

Snack foods^c^	96.9	0.4 (0.1-5.0)	15.7	6.0	1.9
Fruits	99.2	1.0 (0.1-7.0)	43.8	15.0	2.9
Vegetables	99.0	1.0 (0.1-7.0)	29.5	8.0	3.1
Fruits and vegetables	99.2	2.0 (0.3-14.0)	88.5	49.7	28.3

a Among children for whom any consumption was reported (data reported by parents).

b Among all children (data reported by parents).

c Snack foods are chips, pretzels, cookies, or candy. Units for snack foods are times/day.

**Table 3:** Daily Screen Time and Physical Activity Among Children Enrolled in a New York City Women, Infants, and Children (WIC) Program, 2003

**Activity**	**Any Time Spent (%)**	**Median No. of Hours^a^ (Range)**	**Time/Day^b^ (%)**		

			**≥30 min**	**≥1 h**	**≥2 h**
Screen time^c^	97.9	2.0 (0.1-7.0)	83.6	62.3	26.3
Active play and exercise	97.2	0.7 (0.0-12.0)	53.8	33.7	15.6

a Among children who spent any time in these activities (data reported by parents).

b Among all children (data reported by parents).

c Time spent watching television or using a computer.

**Table 4:** Crude and Adjusted Odds of Overweight or Risk of Overweight Among a New York City Women, Infants, and Children (WIC) Population by Sample Characteristics, Food and Beverage Consumption, Screen Time, and Physical Activity, 2003

**Category**	**Overweight or at Risk of Overweight (BMI ≥85th Percentile for Sex and Age), %**	**n = 375-450**		**n = 316**	

		**OR (95% CI)^a^**	***P* Value**	**Adjusted OR (95% CI)^a^**	***P* Value**

**Age, y**					

2	37.4	Ref	—	—	—
3	36.6	0.96 (0.63-1.48)	.87	1.19 (0.70-2.05)	.52
4	4.0	1.11 (0.68-1.83)	.67	1.14 (0.61-2.16)	.68

**Sex**					

Male	38.0	Ref	—	—	—
Female	37.3	0.97 (0.66-1.41)	.94	1.19 (0.74-1.92)	.47

**Race or ethnicity**					

Non-Hispanic black	26.7	Ref	—	—	—
Hispanic or Latino	42.4	2.01 (1.22-3.32)	.006	1.98 (1.08-3.63)	.03
Non-Hispanic White	45.0	2.37 (1.12-5.03)	.02	2.70 (1.06-6.90)	.04
Asian, Non-Hispanic	34.3	1.43 (0.63-3.26)	.39	2.12 (0.81-5.51)	.13

**Family origin**					

United States or Puerto Rico	37.3	0.96 (0.61-1.52)	.96	—	—
Other	38.1	Ref	—	—	—

**Asthma**					

Yes	48.3	1.66 (0.96-2.87)	.09	1.79 (0.85-3.78)	.13
No	36.1	Ref	—	Ref	—

**Milk type**					

Whole or 2%	39.0	0.88 (0.42-1.86)	.89	—	—
1% or fat free	41.9	Ref	—	—	—

**Plain milk, servings/day**					

<3	37.7	1.01 (0.68-1.50)	1.00	—	—
≥3	37.4	Ref	—	—	—

**Flavored milk, servings/day**					

>1	38.6	1.06 (0.68-1.65)	.89	—	—
≤1	37.3	Ref	—	—	—

**Fruit juice, servings/day**					

>1	39.0	1.13 (0.76-1.69)	.61	—	—
≤1	36.0	Ref	—	—	—

**Nonjuice fruit drink, servings/day**					

>1	44.9	1.57 (1.03-2.40)	.046	1.58 (0.94-2.65)	.08
≤1	34.1	Ref	—	Ref	—

**Soda or sweetened iced tea, servings/day**					

Any	37.9	1.04 (0.70-1.54)	.91	—	—
None	36.9	Ref	—	—	—

**Water, servings/day**					

<3	33.6	0.71 (0.48-1.06)	.12	—	—
≥3	41.5	Ref	—	—	—

**Fruits and vegetables, servings/day**					

<5 per day	36.5	0.62 (0.35-1.10)	.13	—	—
≥5	48.1	Ref	—	—	—

**Snack foods (e.g., cookies, chips, candy), times/day**					

≥1	34.3	0.76 (0.52-1.13)	.21	—	—
<1	40.5	Ref	—	—	—

**Screen time (television viewing, computer use), h/day**					

>2	46.8	1.54 (0.99-2.38)	.07	1.74 (1.00-3.04)	.05
≤2	36.5	Ref	—	Ref	—

**Exercise or active play, min/day**					

<30	43.5	1.43 (0.94-2.17)	.12	1.80 (1.10-2.94)	.02
≥30	35.0	Ref	—	Ref	—

**Screen time/exercise ratio**					

More than twice as much screen time as exercise	44.8	1.61 (1.04-2.48)	.04	—	—
Twice as much or less	33.5	Ref	—	—	—

a OR indicates odds ratio; CI, confidence interval; Ref, referent group.
